# *DCC*: a Swiss army knife for structure factor analysis and validation

**DOI:** 10.1107/S1600576716004428

**Published:** 2016-04-18

**Authors:** Huanwang Yang, Ezra Peisach, John D. Westbrook, Jasmine Young, Helen M. Berman, Stephen K. Burley

**Affiliations:** a Research Collaboratory for Structural Bioinformatics Protein Data Bank, Department of Chemistry and Chemical Biology, Center for Integrative Proteomics Research, Rutgers, State University of New Jersey, 174 Frelinghuysen Road, Piscataway, NJ 08854, USA; b Institute for Quantitative BioMedicine, Rutgers, State University of New Jersey, 174 Frelinghuysen Road, Piscataway, NJ 08854, USA; c San Diego Supercomputer Center and Skaggs School of Pharmacy and Pharmaceutical Sciences, University of California, San Diego, La Jolla, CA 92093, USA

**Keywords:** Protein Data Bank, structure factor validation, utility programs, *DCC*

## Abstract

Since 2008, X-ray structure depositions to the Protein Data Bank archive (PDB) have required submission of experimental data in the form of structure factor files. RCSB PDB has developed the program *DCC* to allow worldwide PDB (wwPDB; http://wwpdb.org) biocurators, using a single command-line program, to invoke a number of third-party software packages to compare the model file with the experimental data. *DCC* functionality includes structure factor validation, electron-density map generation and slicing, local electron-density analysis, and residual *B* factor analysis. *DCC* outputs a summary containing various crystallographic statistics in PDBx/mmCIF format for use in automatic data processing and archiving pipelines.

## Abstract 2

*DCC* is a wrapper for third-party software packages to aid in structure factor analysis and validation. As the results are recorded in PDBx/mmCIF format, the output from *DCC* can be used in automatic data pipelines.

## 1. Introduction   

The Protein Data Bank (PDB) is the single global archive of biological structures determined by X-ray crystallography, nuclear magnetic resonance (NMR) and three-dimensional electron microscopy. The archive is managed by the Worldwide PDB collaboration (wwPDB) (Berman *et al.*, 2003 ▸). wwPDB members include the Research Collaboratory for Structural Bioinformatics Protein Data Bank (RCSB PDB) (Berman *et al.*, 2000 ▸), Protein Data Bank in Europe (Velankar *et al.*, 2016 ▸), Protein Data Bank Japan (Kinjo *et al.*, 2012 ▸) and the Biological Magnetic Resonance Bank (Ulrich *et al.*, 2008 ▸).

Prior to 2008, only the atomic coordinate model of the structure was required for PDB archive deposition. Subsequently, submission of experimental data (structure factors for X-ray crystallography, restraints and chemical shifts for NMR) became mandatory (http://www.wwpdb.org/news/news?year=2007#29-November-2007). At this time, numerous individual programs were available to aid in the manipulation and validation of the experimental data relative to the model, but all required expertise and familiarity with the details of each program.

*DCC* was created by RCSB PDB to combine and enable use of these existing programs. Some of the features include structure factor validation, electron-density map calculation, real-space *R* (RSR) calculations, detection and correction of partial *B* factors, and production of cut electron maps and scripts for display in *Jmol* (Hanson, 2010 ▸). The program name, *DCC*, comes from one of these functions and was named for electron-density correlation coefficient. These features are used daily by wwPDB biocurators.

## 2. Methods   

### 2.1. Program function   

*DCC* is a Python wrapper for a number of third-party software programs, including *SFCHECK* (Vaguine *et al.*, 1999 ▸), *PHENIX* (Adams *et al.*, 2002 ▸), *REFMAC* (Murshudov *et al.*, 1996 ▸), *MAPMAN* (Kleywegt *et al.*, 2004 ▸) and *CNS* (Brünger *et al.*, 1998 ▸). Through a command-line interface, *DCC* converts structure factor files from any recognized format, creates the specific input files required for each of these programs and then runs the required programs (Table 1 ▸). *DCC* will also utilize whatever metadata are present in the atomic coordinate model file, including ***TLS*** records and wavelength and twinning information, to produce suitable input data for third-party packages. For instance, a virus structure in which strict non-crystallographic symmetry (NCS) refinement has been used may not include atomic coordinates for the entire asymmetric unit in the model file. In this case, *DCC* will expand the coordinates using the NCS operators for use with third-party programs.

One challenge in producing files for input to refinement programs is how best to represent ligands. Refinement programs require a full definition of all chemical components present in the system, including bond order and connectivity. However, for unreleased components, and prior to processing, such information is not available. Therefore, *DCC* treats all ligands as individual atoms for presentation to the refinement programs.

**Figure 1:**
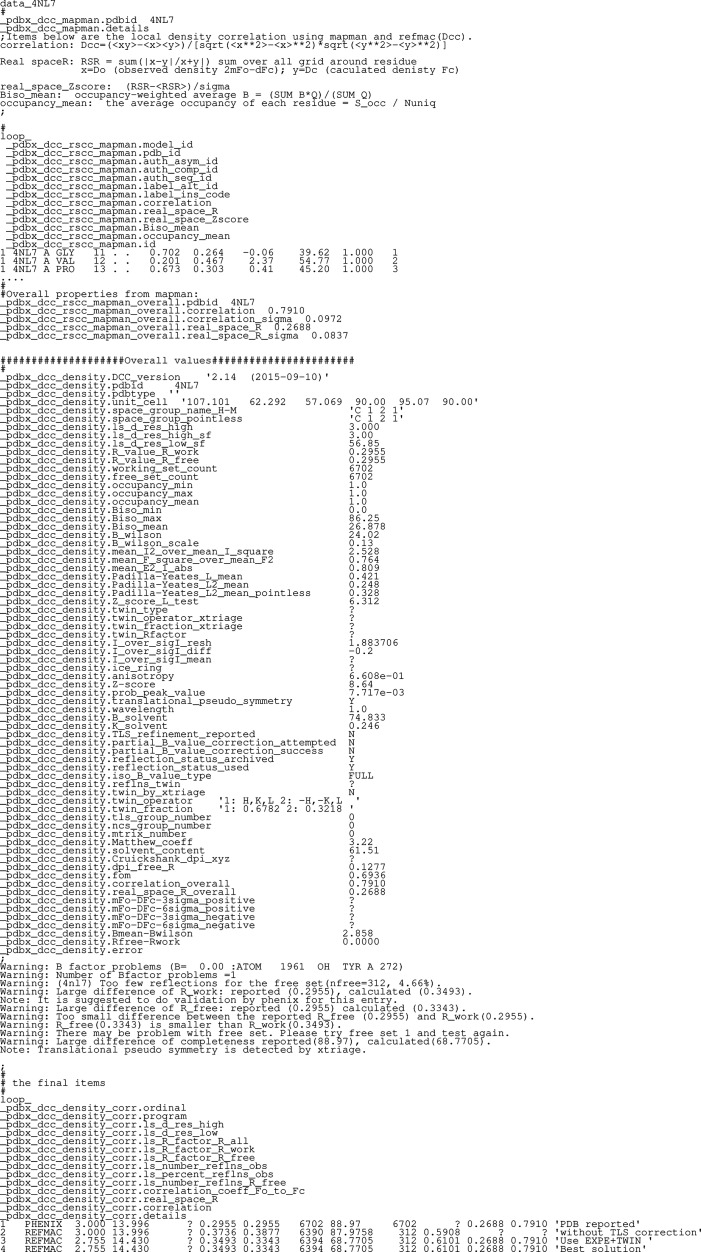
Example output from running *DCC* on the PDB entry 4n7l (Saer *et al.*, 2014 ▸) invoked with the command dcc -pdb 4n7l.cif -sf r4n7lsf.ent using publicly available data from the PDB archive. Ellipses represent sections of the file that have been removed for brevity. The first section is the local real-space density statistics determined by the programs *REFMAC* and *MAPMAN*. The second section is a combination of the model file and the results of phenix.xtriage, and the third section is the result of structure factor validation in *REFMAC*. Various error/warning messages are presented by the PDBx/mmCIF data item (pdbx_density.error). There are more than 200 possible error/warning messages in the *DCC* program. Different structures will export different messages.

For structure factor validation, the user may specify which refinement package to use, or an automatic mode may be invoked that will use the package specified in the model file (Table 1 ▸). A zero-cycle (static) refinement is used and the resulting calculated *R*_work_ and *R*_free_ and other statistics are captured. Based on the statistical analysis of the calculated data items, errors and warnings will be included in the output file. Sample output is depicted in Fig. 1 ▸.

When ***TLS*** restraints are used in refining a structural model with *REFMAC*, authors occasionally deposit structures containing only partial *B* factors without including the isotropic ***TLS*** contribution (Touw & Vriend, 2014 ▸). *DCC* detects these partial *B* factors and then uses *TLSANL* (Howlin *et al.*, 1993 ▸) to produce full *B* factors before performing validation.

*DCC* uses *REFMAC* (Murshudov *et al.*, 1996 ▸) to produce electron-density maps. For local density analysis of both polymer and non-polymer residues, both *EDSTAT* (Tickle, 2012 ▸) and *MAPMAN* (Kleywegt *et al.*, 2004 ▸) are used to calculate RSR factors, density correlations and the real-space difference density *Z* score. *MAPMASK* (Winn *et al.*, 2011 ▸) is used to produce sliced maps for use with *Jmol* visualization.

The results of any analysis, and any additional calculations performed by *DCC*, are captured and stored in a PDBx/mmCIF formatted file. This feature allows *DCC* to be utilized as a component by other programs for further analysis. This capability also allows for the generation of tabular reports for review during PDB archive biocuration and facile loading to relational databases.

## 3. Results and discussion   

The wrapper program *DCC* was developed as a command-line tool that can perform a variety of tasks to aid in the validation of structure factors and atomic coordinate models and the biocuration of PDB depositions. It supports format conversion and generates appropriate input files for a number of third-party programs. By the creation of a simple-to-use front end, biocurators and users are provided access to a variety of software packages without having to know the intricacies of each.

The versatility of a tool such as *DCC* is shown by its use in wwPDB validation reports. In 2008, the wwPDB formed an X-ray Validation Task Force (Read *et al.*, 2011 ▸). To develop validation reports based on their recommendations, the wwPDB created a validation suite for X-ray structures (Gore *et al.*, 2012 ▸) that uses *DCC* to validate deposited structure factors.

Another use case arose during the 2011 wwPDB remediation effort to identify X-ray structures in which partial *B* factors were present in the atomic coordinate model file. Based on the output of *DCC*, annotators corrected ***TLS*** information in the entries and furnished an indicator that only partial *B* factors were present.

## 4. Conclusions   

The program *DCC* is a versatile tool that is used daily by wwPDB biocurators. The usage of PDBx/mmCIF allows *DCC* to be employed in automatic pipelines. It is available for download from http://sw-tools.rcsb.org.

## Figures and Tables

**Table 1:** The list of command-line options available in *DCC* The basic command ‘dcc -pdb xyzfile -sf sffile’ performs the default functionalities described in the text using *REFMAC*. Any metadata in the model file are utilized in the calculations. If such information is not found in the file, parameters are optimized so that the calculated statistics best match those reported.

The basic command:	
dcc -pdb xyzfile -sf sffile	Where xyzfile is the coordinate file in either PDB or PDBx(mmCIF) format, and sffile is the structure factor file, which can be in any of the following formats [mtz, mmCIF, CIF (for small molecules; IUCr), *CNS/Xplo*r, *HKL2000*/*SCALEPACK*, *Dtrek*, *SHELX*, *SAINT*, *EPMR*, *XSCALE*, *XPREP*, *TNT*, *XTALVIEW*, *X-GEN*, *XENGEN*, *MULTAN* and *MAIN*].


The options below can be added to the above command to perform additional tasks:	
-o	Followed by an output file name to hold the calculated statistics. If not given, the default name (pdbfile + _rcc_sum.cif) will be used.
-diags	Followed by a log file name to hold error/warning messages.
-verb	Add to keep the intermediate files during computations.
-rsr_all	Add to calculate electron-density statistics (RSR, RSRZ, RSCC) by groups [residual, main chain, side chain, phosphate (if RNA/DNA)].
-edstat	Add to use the *EDSTAT* program to calculate electron-density statistics (RSR, RSRZ, RSCC, RSZD, RSDO) by groups [residual, main chain, side chain, phosphate (if RNA/DNA)].
-sfcheck	Add to validate X-ray data by *SFCHECK*.
-refmac	Add to validate X-ray data by *REFMAC* (default).
-phenix_x	Add to validate X-ray data by *PHENIX* (model_vs_data).
-phenix_n	Add to validate neutron data by *PHENIX* (model_vs_data).
-phenix_xn	Add to validate neutron and X-ray hybrid data by *PHENIX* (model_vs_data). The structure factor file (sffile) must be in mmCIF format. The first data block must be the X-ray data and the second data block must be the neutron data.
-cns	Add to validate X-ray data by *CNS*/*Xplor*.
-all	Add to validate X-ray data by all the programs (*SFCHECK*, *REFMAC*, *Phenix*). The calculated statistics such as *R*/*R*_free_ will be listed by the programs.
-auto	Add to validate X-ray data by the program used for refinement in the coordinate file (xyzfile). If the program fails then other programs will be used.
-map	Add to calculate maps (*mF*_o_ − *DF*_c_, *2mF*_o_ − *DF*_c_) in *CCP4* format.
-ligmap	Add to produce all the files (ligand density maps, tables and html files) and *Jmol* scripts for displaying the ligand density in a browser.
-omitmap	Add to calculate residual electron-density statistics (RSR, RSRZ, RSCC) after omitting all the ligands.
-omit	Followed by an identifier to calculate the omit map. For example, the command dcc -pdb xyzfile -sf sffile -omit A_3:5 calculates a map omitting residue numbers from 3 to 5 of chain *A*.
-fem	Add to calculate density statistics and the map using the feature-enhanced map in *PHENIX*.
-bfull	Convert residual to full *B* factors using the command dcc -bfull xyzfile.
